# A herb mixture to ameliorate non-alcoholic fatty liver in rats fed a high-fat diet

**DOI:** 10.1016/j.heliyon.2023.e18889

**Published:** 2023-08-02

**Authors:** Sang Keun Ha, Jin-Ah Lee, Donghwan Kim, Guijae Yoo, Inwook Choi

**Affiliations:** a Food Functionality Research Division, Korea Food Research Institute, Wanju-gun, Republic of Korea; b Division of Food Biotechnology, University of Science and Technology, Daejeon, South Korea

**Keywords:** Non-alcoholic fatty liver disease, *Zingiber officinale*, *Centella asiatica*, *Boehmeria nivea*, Combinatorial herb extract

## Abstract

This study was performed to investigate the effects of an herb extract mixture (HM) in ameliorating non-alcoholic fatty liver disease (NAFLD). The HM contained equal amounts of 70% ethanol extracts from *Zingiber officinale*, *Centella asiatica*, and *Boehmeria nivea*. *In vitro*, the HM significantly inhibited lipid accumulation in oleic acid-stimulated HepG2 cells. We further evaluated the *anti*-NAFLD activities of the HM *in vivo* in an animal model. Rats were fed two different amounts of the HM (50 and 200 mg/kg body weight) along with a high-fat diet for 6 weeks. HM supplementation reduced liver weight; epididymal, peri-renal, and intra-abdominal fat content; and serum triglyceride, total cholesterol, and low-density lipoprotein cholesterol levels as well as increased high-density lipoprotein cholesterol levels in a dose-dependent manner. Histological evaluation of liver specimens further demonstrated that administration of HM significantly prevented hepatic lipid accumulation and subsequent development of hepatic steatosis. These findings suggest that HM can be used as an alternative nutraceutical for ameliorating NAFLD.

## 1 Introduction

Non-alcoholic fatty liver disease (NAFLD) is a condition associated with the accumulation of lipids in the liver and can progress into simple steatosis, steatohepatitis, fibrosis, cirrhosis, and hepatocellular carcinoma [1,2]. Global NAFLD prevalence and incidence are higher than previous estimated [3]. Especially, NAFLD prevalence is rapidly increasing in certain subpopulations such as obese and metabolic syndrome [4]. Several molecular and metabolic changes with the ‘multiple hit’ such as insulin resistance, hormones, nutritional factors and gut microbiota take place in NAFLD [5]. Current study showed that anti-diabetic agents, glucagon-like peptide-1 modulators, sodium-glucose transport protein inhibitors, and thiazolidinedione insulin sensitizers can be potentially used to NAFLD medicine [6]. Modulation of glucose and lipid metabolism by natural products alleviated NAFLD [7,8]. However, there is no FDA approved the medicine for NAFLD treatment. Development of medicine for NAFLD is urgently needed.

Development of a single herb extract for treating certain diseases has been the main focus of research and development to date [[9], [10], [11], [12]]. *Zingiber officinale* (the rhizomes of ginger) is a herbal medicine used to relieve inflammation, high cholesterol (hypercholesterolemia), obesity, and diabetes [13,14]. Ginger treatment was shown to significantly decrease the levels of both serum cholesterol and triglycerides in mice [15]. *Centella asiatica* is Asian traditional herb that has protection role from hepatotoxicity through inhibition of oxidative stress, inflammation and apoptosis [16]. *Centella asiatica* was also reported to ameliorate early stage of hyperlipidemia in rats [17]. *Boehmeria nivea* has been used for its anti-inflammatory and anti-oxidative properties [18,19].

Herb mixtures (HM) is useful approach to development therapeutic agents [20,21]. We previously explored the functionality of herbs by evaluating changes in gene expression profiles before and after herb extract addition to various cell lines. This system is unique in terms of inferring functionalities of herb mixtures compared to typical research which focuses on the genetic effects of individual herb extracts. A prerequisite for selecting an appropriate herb mixture is that it is expected to have better functionality than the individual herb components based on the functionality inferring system. In this study, we investigated the effect of *Zingiber officinale*, *Centella asiatica*, and *Boehmeria nivea* mixture on hepatic lipid accumulation using HepG2 cells and in NAFLD rat models.

## 2 Materials and methods

### 2.1 Preparation of herbal extracts

The three herbs, *Z. officinale*, *C. asiatica*, and *B. nivea*, were purchased from DAMAONYAKCHO (Yeongcheon-si, Gyeongsangbuk-do, Republic of Korea). To prepare the extracts from 100 g of pulverized powder, each powder was suspended in 2 L of 70% aqueous ethanol and ultrasonicated for 1 h in a 750-W ultrasonic processor (VCX 750, Sonics and Materials, Inc., Newtown, CT, USA). Extraction in the ultrasonic processor was repeated three times. The undissolved residue was filtered out using quantitative Whatman No. 1 filter paper (Whatman, Maidstone, UK) and centrifugation. The extracts were evaporated on a rotary vacuum evaporator (R205, Buchi, Fostfach Switzerland) and lyophilized to yield a dry powder.

### 2.2 Cell culture

Human hepatoblastoma HepG2 cells were obtained from American Type Culture Collection (ATCC; Manassas, VA, USA) and cultured in Dulbecco's modified Eagle medium (DMEM; Hyclone, Logan, UT, USA) supplement with 1% penicillin/streptomycin (Gibco, Grand Island, NY, USA), and 10% fetal bovine serum (FBS, ATCC) at 37 °C in a humidified atmosphere of 5% CO_2_ in air.

### 2.3 Cell viability assay

For cell viability tests following exposure to oleic acid (OA) and herbal extracts, HepG2 cells were seeded at a density of 5 × 10^4^ cells/well in 96-well plates and incubated with various concentrations of OA (Sigma-Aldrich, St. Louis, MO, USA) or herbal extracts in 1% bovine serum albumin (BSA, Sigma, USA)-supplemented DMEM for 24 h. The medium containing only 1% BSA was used as the control. After incubation, the medium was discarded, and 0.1 mg/ml MTT reagent was added and incubated for 4 h. The solution was removed, and dimethyl sulfoxide was added to dissolve the formazan crystals. The absorbance of the solution at 570 nm was measured with a spectrophotometer (Molecular Devices, Sunnyvale, CA, USA).

### 2.4 ORO staining

HepG2 cells were seeded at a density of 2 × 10^5^ cells/well in 24-well plates and incubated with OA (0.6 mM) and/or individual herbs (*Z. officinale*, *C. asiatica*, *B. nivea*; 100 μg/ml) or the combinational herb mixture (HM; 50 or 100 μg/ml) in 1% BSA-supplemented DMEM for 24 h. An equal proportion of extracts obtained from *Z. officinale*, *C. asiatica*, and *B. nivea* was mixed and used as the HM. After washing the cells twice with Dulbecco's phosphate-buffered saline, they were fixed with 10% formalin for 30 min. The cells were washed again with Dulbecco's phosphate-buffered saline and stained with a freshly prepared solution of ORO (Sigma-Aldrich) for 15 min at room temperature in the dark. After washing several times, the cells were observed under a microscope (Nikon, Tokyo, Japan). To quantify the ORO content, isopropanol was added to each sample followed by shaking at room temperature for 10 min. The optical density of the isopropanol-extracted sample was measured with a spectrophotometer at 510 and 630 nm.

### 2.5 Animal model of NAFLD

Seven-week-old male Sprague-Dawley rats (n = 32, 280 ± 10 g) were purchased from Orient (Seungnam, Korea). The animals were kept under controlled environmental conditions (22 ± 3 °C with a 12/12-h light/dark cycle) for one week prior to the experiment. The rats were divided into the following four groups (n = 8 per group): ND, HFD, HFD + 50 mg/kg HM (HM50), and HFD + 200 mg/kg HM (HM200). An equal proportion of extracts obtained from *Z. officinale*, *C. asiatica*, and *B. nivea* was mixed and used as the HM. All rats were provided food and water *ad libitum*. All groups except the ND (Teklad rodent diet 2918C; ENVIGO, Barcelona, Spain; 5.77% fat, 44.2% carbohydrate, 17.7% protein) were provided a 60% fat calorie diet (Teklad Custom diet TD.06414; ENVIGO, Barcelona, Spain; 60.3% fat, 21.4% carbohydrate, 18.3% protein). The HM was orally administered daily for 6 weeks to the two HM groups along with the HFD. Feed intake and body weight were measured weekly for 6 weeks. At the end of the experimental period, the animals were fasted for 14 h prior to anesthetization with intraperitoneal injection of ketamine (80 mg/kg) and xylazine (8 mg/kg) and sacrifice. The blood from abdominal aorta (800 μl), liver, and parametrial adipose tissues (epididymal fat, peri-renal fat, and intra-abdominal fat) were quickly removed and measured weight. All animal studies were performed in accordance with international regulations of the usage and welfare of laboratory animals and protocols approved by the Institutional Animal Care and Use Committee in Chonbuk National University Hospital in terms of ethic procedures and scientific care (IACUC, cuh-IACUC-2017-18).

### 2.6 Biochemical analysis

The collected blood was left at room temperature for 30 min and then centrifuged at 3000 rpm for 10 min to obtain the serum. The serum TG and TC levels were measured with commercial enzymatic assay kits from Asan Pharmaceutical Co. (#AM202, Seoul, Korea), and HDL and LDL levels were measured using commercial enzymatic assay kits from BioVision (#K613-100, Milpitas, CA, USA).

### 2.7 Histopathological examination

Harvested livers and eWAT were fixed in 10% formalin. After fixation, the tissues were washed in tap water and then paraffinized. Paraffinized tissues were embedded in a paraffin block then cut into 5 μm thick sections with a rotary microtome. The sections were deparaffinized in xylene, rehydrated through a descending alcohol series (100%, 95%, 90%, 80%, and 70%), and hydrated sections were washed in PBS. After removing PBS by wiping, the sections were stained with Harris’ hematoxylin for 5 min and eosin for 3 min. The sections were mounted using a cover glass and DPX Mountant for histological analysis. The mounted sections were imaged using an upright microscope (Olympus, Tokyo, Japan). The diameter of cells (n = 100 cells) was measured from four sections for each group and analyzed using ImageJ software (NIH, Bethesda, MD, USA).

### 2.8 Analytical method condition

Analysis was performed on the High-performance liquid chromatography (HPLC) system (Waters Corporation, Milford, MA, USA). The HPLC system was equipped with a Waters Alliance e2695 Separations Module and UV/VIS detector (Waters, 2489). The output signal of the detector was recorded using a Empower® 3 software. The separation was executed on a YMC Hydrosphere C_18_ column (12 nm, 150 × 4.6 mm, 5 μm). The mobile phase was composed of 0.1% formic acid and acetonitrile with the gradient elution system at a flow rate of 0.4 ml/min. The gradient flows were as follows: 10–35% B at 0–8 min, 35–45% B at 8–20 min, 45–70% B at 20–25 min, 70–90% B at 25–35 min, 90% B at 35–40 min. The injection volume was 10 μl and the detection wavelength was set at 205 nm.

### 2.9 Statistical analysis

Data are expressed as the means ± standard error of the mean for the number of replicates indicated. The significance of differences between groups was assessed by one-way ANOVA with Tukey correction using Prism 7.0 software (GraphPad Software, La Jolla, CA, USA).

## 3 Results

### 3.1 Effect of HM on lipid accumulation in HepG2 cells

**Fig. 1:**
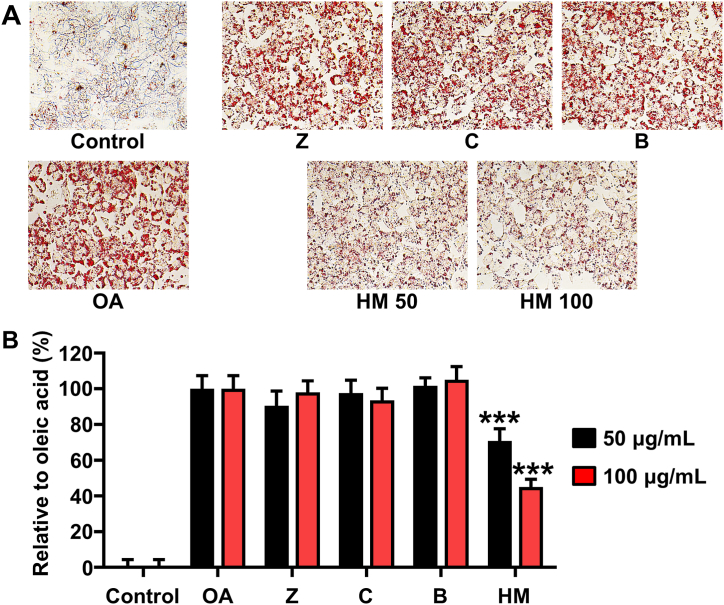
Effect of the herb extract mixture (HM) on lipid accumulation in HepG2 cells. The cells were treated with individual herbs (*Zingiber officinale* [Z], *Centella asiatica* [C], *Boehmeria nivea* [B]; 50 and 100 μg/ml) or their combination (HM; 50 and 100 μg/ml) in the presence of oleic acid (OA; 0.6 mM) for 24 h, and stained with Oil Red O. (A) Lipid droplets observed by microscopy. (B) Quantitative assessment of the percentage of lipid accumulation based on Oil Red O staining. Data are expressed as the mean ± SD. ^###^*p* < 0.001 compared to the OA group.

We used OA to prepare an *in vitro* model of NAFLD in HepG2 cells to explore the potential lipid-lowering effects of the HM. The cytotoxicity of OA and HM was first assessed. OA and HM did not affect the viability of HepG2 cells at up to 0.6 mM and 200 μg/ml, respectively, based on the results of MTT assays (data not shown). To verify the inhibition of OA-induced lipid accumulation by HM or the individual herb extracts, HepG2 cells were treated with 100 μg/ml concentrations of individual herb extracts or the HM in the presence of OA (0.6 mM) for 24 h. The cells were then stained with ORO, and lipid accumulation was quantified by measuring the absorbance at 510 nm. HM reduced the OA-induced ORO signal in a dose-dependent manner (Fig. 1A). Specifically, lipid accumulation was reduced by 30% and 55% at HM concentrations of 50 and 100 μg/ml, respectively (Fig. 1B). Whereas the individual herbs *Z. officinale*, *C. asiatica*, and *B. nivea* did not show any inhibitory activity on OA-induced lipid accumulation in HepG2 cells (Fig. 1B).

### 3.2 Effects of HM on the body fat index of rats

**Fig. 2:**
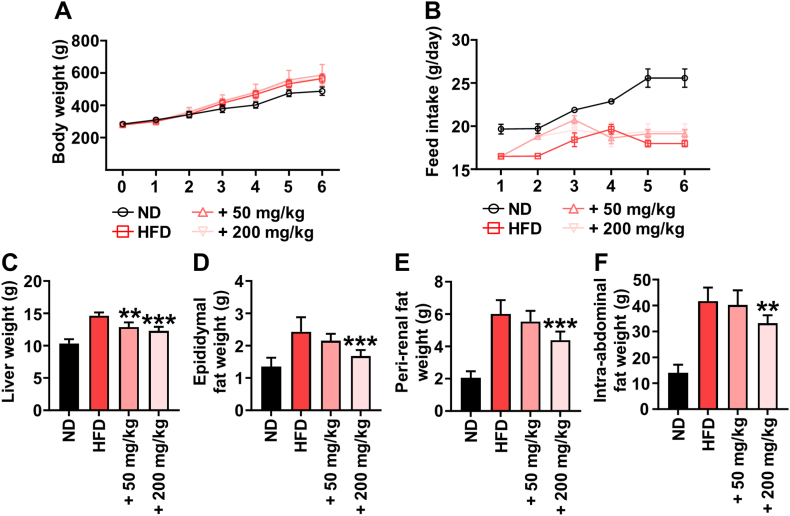
Effect of combinatorial herb mixture (HM) on the weights of the liver, epididymal fat, peri-renal fat, and intra-abdominal fat tissues in rats. (A) Body weight and (B) feed intake. (C) Liver weight (D) epididymal fat weight (E) peri-renal fat weight and (F) intra-abdominal fat weight. Data are expressed as the mean ± SD (n = 8). ^##^*p* < 0.01; ^###^*p* < 0.001 compared to the high-fat diet (HFD) group.

To further investigate the ameliorating activity of HM against NAFLD, HM was supplemented to rats fed HFD. After 6 weeks of HM supplementation, the liver, epididymal fat, peri-renal fat, and intra-abdominal fat tissues were harvested from the rats, and their weights were measured. There were no significant differences in feed intake and body weights between the HFD and HM groups after 6 weeks (Fig. 2A and B). However, the weights of the epididymal fat, peri-renal fat, and intra-abdominal fat tissues were significantly decreased in the HM group compared to those in the HFD group (Fig. 2C–F).

### 3.3 Effects of HM on serum lipid profiles of rats

**Fig. 3:**
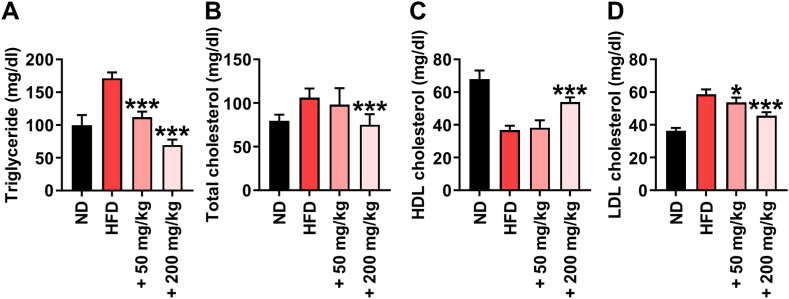
Effect of a combinatorial herb mixture (HM) on (A) serum triglyceride (B) total cholesterol (C) high-density lipoprotein (HDL)-cholesterol and (D) low-density lipoprotein (LDL)-cholesterol levels in rats. Data are expressed as mean ± SD (n = 8). ^#^*p* < 0.05; ^###^*p* < 0.001 compared to the high-fat diet (HFD) group.

Treatment with HM caused a significant decrease in serum TG, TC, and LDL levels of rats fed the HFD (Fig. 3A–D). The levels of serum TG in both HM groups (50 and 200 mg/kg) were significantly lower than those in the HFD group, whereas only 200 mg/kg HM resulted in a significant decrease in the serum TC level (Fig. 3A and B). The HFD caused a significant decrease in HDL levels after 6 weeks, whereas daily co-supplementation with 200 mg/kg of HM alleviated HFD-induced HDL reduction. Both HM groups (50 and 200 mg/kg) showed a significant reduction in serum LDL compared to that in the HFD group after 6 weeks of feeding (Fig. 3C and D). These results indicate that supplementation of HM helps to maintain a normal serum lipid profile under HFD feeding.

### 3.4 Effects of HM on histology of rat tissues

**Fig. 4:**
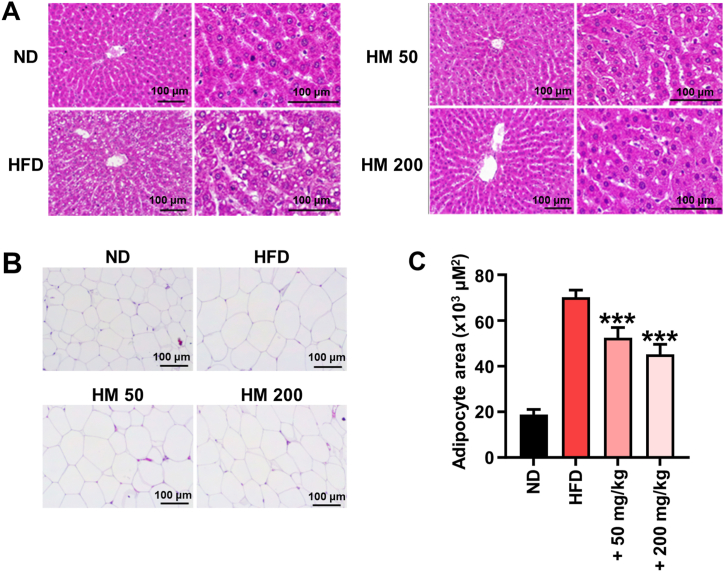
Effect of a combinatorial herb extracts mixture (HM) on histological changes in high-fat diet (HFD)-induced non-alcoholic fatty liver disease (NAFLD). (A) Liver and (B) epididymal white adipose tissue (eWAT) stained with hematoxylin and eosin and observed microscopically at 100 × and 400 × magnification. (C) Adipocyte area in the eWAT measured using ImageJ software. ND: normal diet; HFD: high-fat diet; HM 50: HFD +50 mg/kg herb mixture; HM 200: HFD +200 mg/kg herb mixture. Data are expressed as the mean ± SD (n = 8). ^###^*p* < 0.001 compared with the HFD group.

To examine whether the reductions in liver weight was attributed to decreased hepatic lipid accumulation, the liver samples were fixed, sectioned, and stained with hematoxylin and eosin. The staining results revealed that lipid droplet in the HFD group was observed, whereas HFD-induced lipid droplet was hindered by HM supplementation (Fig. 4A). The HFD group also displayed a significantly increased eWAT mass and adipocyte area than the ND-fed group, which was also significantly suppressed by supplementation with both 50 and 200 mg/kg body weight/day of HM (Fig. 4B). These data suggest that supplementation of HM prevents HFD-induced lipid accumulation in liver and eWAT.

### 3.5 Analytical method development and validation

**Fig. 5:**
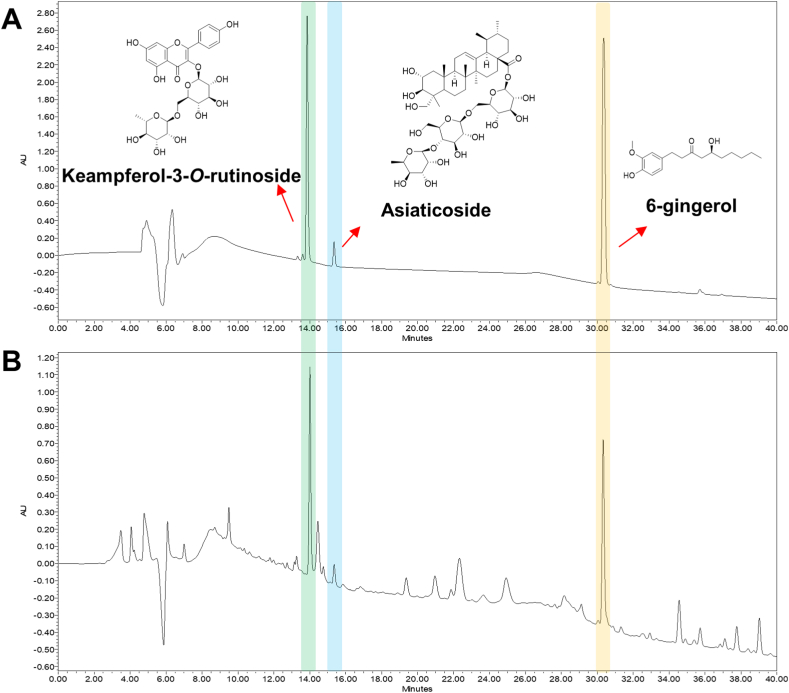
The high-performance liquid chromatography (HPLC) chromatogram of standard solution and three-herb mixture extracts. (A) HPLC chromatograms of standard solution. (B) HPLC chromatograms of three-herb mixture extracts.

A simple and reliable analytical method has been developed and validated for simultaneous determination of the three major components (kaempferol-3-*O*-rutinoside, asiaticoside, 6-gingerol) in herb mixture (Fig. 5A and B). Method validation was executed by linearity, precision and accuracy test on the basis of ICH guidelines [22]. The calibration curve of three components showed good linearity (R^2^ > 0.9962) (Table 1). The limit of detection (LOD) and limit of quantification (LOQ) were observed within the ranges 0.013 to 0.07 and 0.04–0.21 μg/μL, respectively. The relative standard deviation (RSD) values of intra- and inter-day testing were indicated that less than 3%. The results of recovery test were 96.28–99.99% and RSD range was measured from 0.03 to 1.74% (Table 2). In conclusion, this analytical method has been successfully applied to the simultaneous determination of three components in herb mixture.

**Table 1:** Analytical results of linearity, limit of detection, and limit of quantification.

Analyte	Regression equation	Correlation coefficient (R^2^)	Linear range (μg/μl)	LOD^a^ (μg)	LOQ^b^ (μg)
Kaempferol-3-*O*-rutinoside	y = 6,599,589x + 507,847	0.9987	0.02–0.333	0.0127	0.0384
Asiaticoside	y = 565,293× - 9604	0.9995	0.02–0.333	0.0689	0.2087
6-Gingerol	y = 10,581,395x + 1,913,396	0.9962	0.02–0.333	0.0322	0.0977

LOD^a^: limit of detection.

LOQ^b^: limit of quantification.

**Table 2:** Analytical results of intra-, inter-day tests and recovery test of three major components in the herb mixture extracts.

Analyte	Precision RSD (%)				Accuracy RSD (%)				
	Concentration (μg/mL)	Intra-day (n = 3)	Inter-day (n = 3)	Repeatability RSD (%) (n = 5)	Sam:Std	Spiked (μg)	Detected (μg)	Recovery (%)	RSD (%)
Kaempferol-3-*O*-rutinoside	0.333	0.1738	0.7734	0.6389	1 : 2	2.0135	1.9841	98.5400	0.6658
	0.166	0.4676	0.3479		1 : 1	1.7702	1.7044	96.2796	0.0966
	0.083	0.6189	0.6933		2 : 1	1.5270	1.5022	98.3805	1.7386
Asiaticoside	0.333	1.9641	2.9759	0.3698	1 : 2	2.1770	2.1460	98.5767	0.3534
	0.166	0.6862	1.6840		1 : 1	2.0155	2.0135	99.8979	0.0535
	0.083	2.4716	2.3285		2 : 1	1.8541	1.8076	97.4934	0.8276
6-Gingerol	0.333	0.3770	0.7848	0.7326	1 : 2	1.8702	1.8514	99.9962	0.9726
	0.166	0.8517	1.0181		1 : 1	1.5553	1.5435	99.2388	0.0269
	0.083	0.9772	1.0740		2 : 1	1.2404	1.2283	99.0267	1.3719

## 4 Discussion

There is many studies to develop the NAFLD drugs using single herb extract [23,24]. *Z. officinale*, *C. asiatica*, and *B. nivea* have anti-inflammatory effects in various inflammatory diseases respectively [18,25,26]. However, single dose treatment of each of these are not significantly suppressed lipid accumulation in HFD-induced NAFLD rat model in our previous study (data not shown). In this case, herb mixtures may be more useful as therapeutic approach. In the present study, we evaluated effect of herbal mixture from *Z. officinale*, *C. asiatica*, and *B. nivea* on NAFLD rat model. Treatment of HM suppressed OA-induced lipid accumulation in HepG2 cells and HFD-induced lipid droplet in NAFLD rat model. Especially, HM treatment improved HFD-induced body fat index in NAFLD model.

NAFLD is the most common form of chronic liver disease in parallel with the obesity characteristic, with the fastest emerging manifestations of the metabolic syndrome worldwide [27]. Multiple hits by insulin resistance, hormones and epigenetic factors in fatty liver accelerate NAFLD progression [5]. Current study showed that gut microbiota alternation causes NAFLD [28]. Progressive form of NAFLD, nonalcoholic steatohepatitis, leads to cirrhosis and liver cancer [29]. Accumulating evidence indicates that NAFLD patients are at potential risk for the development of hypertension, coronary heart disease, and cardiomyopathy that clinically result in increased cardiovascular mortality [30]. Many studies showed that herbal extracts have suppression role for NAFLD [28]. However, knowledge on the pathogenesis and therapeutic strategy of NAFLD is still unclear.

OA is not an essential fatty acid that is endogenously synthesized by Stearoyl-CoA desaturase 1 in humans [31]. Excessive accumulation of OA through *de novo* fatty acid synthesis leads to lipogenesis and NAFLD in HepG2 cells [32,33]. Treatment of HepG2 cells with OA induced morphological changes similar to steatotic characteristics [34]. Single treatment of herbal extract from *Z. officinale*, *C. asiatica*, or *B. nivea* not suppressed OA-induced lipid accumulation in HepG2 cells. However, HM treatment suppressed OA-induced lipid accumulation in HepG2 dose dependently. *Z. officinale* and *C. asiatica* have *anti*-hyperlipidemic activity [35,36]. Especially *Z. officinale* suppressed 3% cholesterol and 15% butter containing diet induced SCD1 expression in rodent liver [35]. These results indicate that HM may suppress OA-induced lipid accumulation through SCD1 suppression and is a potent nutraceutical which may useful for treating NAFLD.

The HFD-induced NAFLD rat model has been widely used for evaluating the pharmacological effects of drugs on NAFLD [37,38]. HFD increased TC, LDL, and TAG levels and suppressed HDL levels in rodent model [[39], [40], [41]]. HFD-induced NAFLD model is more accurately reflects the human clinical NAFLD pathologies, including obesity, insulin resistance, and high serum triglycerides as well as the typical hepatic steatosis, and inflammation [42]. Thus, this model was used for further study to validate the effects of HM on NAFLD. Liver histology analysis showed that HFD induced typical steatosis based on ballooning degeneration and numerous lipid droplets. Adipose tissue mass and dyslipidemia were increased by HFD. This is mainly attributed to adipocyte hypertrophy caused by increased levels of serum TG, TC, and free fatty acids. Supplementation of the HM effectively suppressed HFD-induced body fat indices such as liver weight, peri-renal fat weight, intra-abdominal fat weight and epididymal fat weight induction. HFD-induced serum TG, TC, and LDL induction ameliorated by HM supplementation. HFD-induced lower HDL levels were recovered by HM supplementation. Administration of bioactive compound from natural product reduced HFD-induced TG, TC, and LDL levels and ameliorated HDL levels [43,44]. *Z. officinale*, *C. asiatica*, and *B. nivea* suppress lipid accumulation, blood glucose levels, and fat accumulation in liver respectively [[45], [46], [47]]. Therefore, HM supplementation may improve lipid profiles against NAFLD. Histological observation of liver specimens also demonstrated that HFD-induced lipid accumulation and development of hepatic steatosis were suppressed by HM supplementation. These results indicate that HM have *anti*-hypolipidemic activity which suppressed HFD-induced NAFLD model.

HPLC analysis revealed that keampferol-3-*O*-rutinoside, asiaticoside and 6-gingerol were identified as major compound in HM. Keampferol-3-*O*-rutinoside has *anti*-adipogenesis activity [48]. Asiaticoside have protective effects on acute liver injury [49]. 6-Gingerol suppressed HFD-induced NAFLD through activating LKB1/AMPK pathways in mice [50]. These results indicate that HM containing each compounds may suppress HFD-induced NAFLD in rat.

In conclusion, present study has limitation about action mechanism of HM. However, HM treatment markedly suppressed HFD-induced pathological changes in both HepG2 cells and the HFD-induced NAFLD rat model. These data demonstrate that the HM from *Z. officinale*, *C. asiatica*, and *B. nivea* may a potent nutraceutical for alleviating NAFLD.

## Ethics statement

All animal studies were performed in accordance with international regulations of the usage and welfare of laboratory animals and protocols approved by the Institutional Animal Care and Use Committee in Chonbuk National University Hospital in terms of ethic procedures and scientific care (IACUC, cuh-IACUC-2017-18).

## Author contribution statement

Sang Keun Ha, Guijae Yoo: Performed the experiments; Analyzed and interpreted the data.

Jin Ah Lee: Performed the experiments; Analyzed and interpreted the data; Wrote the paper.

Donghwan Kim: Analyzed and interpreted the data; Wrote the paper.

Inwook Choi: Conceived and designed the experiments; Analyzed and interpreted the data.

## Data availability statement

Data included in article/supplementary material/referenced in article.

## Declaration of competing interest

The authors declare that they have no known competing financial interests or personal relationships that could have appeared to influence the work reported in this paper.

## References

[1] Minaya D.M., Turlej A., Joshi A., Nagy T., Weinstein N., DiLorenzo P., Hajnal A., Czaja K. (2020). Consumption of a high energy density diet triggers microbiota dysbiosis, hepatic lipidosis, and microglia activation in the nucleus of the solitary tract in rats. Nutr. Diabetes.

[2] Mooli R.G.R., Ramakrishnan S.K. (2022). Emerging role of hepatic ketogenesis in fatty liver disease. Front. Physiol..

[3] Riazi K., Azhari H., Charette J.H., Underwood F.E., King J.A., Afshar E.E., Swain M.G., Congly S.E., Kaplan G.G., Shaheen A.A. (2022). The prevalence and incidence of NAFLD worldwide: a systematic review and meta-analysis. Lancet Gastroenterol Hepatol.

[4] Henry L., Paik J., Younossi Z.M. (2022). Review article: the epidemiologic burden of non-alcoholic fatty liver disease across the world. Aliment. Pharmacol. Ther..

[5] Ore A., Akinloye O.A. (2021). Phytotherapy as multi-hit therapy to confront the multiple pathophysiology in non-alcoholic fatty liver disease: a systematic review of experimental interventions. Medicina (Kaunas).

[6] Ferguson D., Finck B.N. (2021). Emerging therapeutic approaches for the treatment of NAFLD and type 2 diabetes mellitus. Nat. Rev. Endocrinol..

[7] Shao G., Liu Y., Lu L., Zhang G., Zhou W., Wu T., Wang L., Xu H., Ji G. (2022). The pathogenesis of HCC driven by NASH and the preventive and therapeutic effects of natural products. Front. Pharmacol..

[8] Yang S., Cao S., Li C., Zhang J., Liu C., Qiu F., Kang N. (2022). Berberrubine, a main metabolite of berberine, alleviates non-alcoholic fatty liver disease via modulating glucose and lipid metabolism and restoring gut microbiota. Front. Pharmacol..

[9] Mun J., Kim S., Yoon H.G., You Y., Kim O.K., Choi K.C., Lee Y.H., Lee J., Park J., Jun W. (2019). Water extract of curcuma longa L. Ameliorates non-alcoholic fatty liver disease. Nutrients.

[10] Li X., Zhang W., Liang L., Duan X., Deng J., Zhou Y. (2020). Natural product-derived icaritin exerts anti-glioblastoma effects by positively modulating estrogen receptor beta. Exp. Ther. Med..

[11] Simental-Mendia L.E., Gamboa-Gomez C.I., Guerrero-Romero F., Simental-Mendia M., Sanchez-Garcia A., Rodriguez-Ramirez M. (2021). Beneficial effects of plant-derived natural products on non-alcoholic fatty liver disease. Adv. Exp. Med. Biol..

[12] Zhu J., Ding J., Li S., Jin J. (2022). Ganoderic acid A ameliorates non-alcoholic streatohepatitis (NASH) induced by high-fat high-cholesterol diet in mice. Exp. Ther. Med..

[13] Kondapalli N.B., Hemalatha R., Uppala S., Yathapu S.R., Mohammed S., Venkata Surekha M., Rajendran A., Bharadwaj D.K. (2022). Ocimum sanctum, Zingiber officinale, and Piper nigrum extracts and their effects on gut microbiota modulations (prebiotic potential), basal inflammatory markers and lipid levels: oral supplementation study in healthy rats. Pharm. Biol..

[14] Salaramoli S., Mehri S., Yarmohammadi F., Hashemy S.I., Hosseinzadeh H. (2022). The effects of ginger and its constituents in the prevention of metabolic syndrome: a review. Iran J Basic Med Sci.

[15] Tometsuka C., Funato N., Mizuno K., Taga Y. (2021). Long-term intake of ginger protease-degraded collagen hydrolysate reduces blood lipid levels and adipocyte size in mice. Curr. Res. Food Sci..

[16] Ferah Okkay I., Okkay U., Aydin I.C., Bayram C., Ertugrul M.S., Mendil A.S., Hacimuftuoglu A. (2022). Centella asiatica extract protects against cisplatin-induced hepatotoxicity via targeting oxidative stress, inflammation, and apoptosis. Environ. Sci. Pollut. Res. Int..

[17] Setyaningsih W.A.W., Arfian N., Fitriawan A.S., Yuniartha R., Sari D.C.R. (2021). Ethanolic extract of Centella asiatica treatment in the early stage of hyperglycemia condition inhibits glomerular injury and vascular remodeling in diabetic rat model. Evid Based Complement Alternat Med.

[18] Lim J.Y., Lee J.H., Lee B.R., Kim M.A., Lee Y.M., Kim D.K., Choi J.K. (2020). Extract of Boehmeria nivea suppresses mast cell-mediated allergic inflammation by inhibiting mitogen-activated protein kinase and nuclear factor-kappaB. Molecules.

[19] Lee H.J., Choi E.J., Park S., Lee J.J. (2020). Laxative and antioxidant effects of ramie (Boehmeria nivea L.) leaf extract in experimental constipated rats. Food Sci. Nutr..

[20] Cho B.O., Shin J.Y., Kang H.J., Park J.H., Hao S., Wang F., Jang S.I. (2021). Antiinflammatory effect of Chrysanthemum zawadskii, peppermint, Glycyrrhiza glabra herbal mixture in lipopolysaccharidestimulated RAW264.7 macrophages. Mol. Med. Rep..

[21] Pak P.J., Lee D.G., Sung J.H., Jung S.H., Han T.Y., Park S.H., Chung N. (2021). Synergistic effect of the herbal mixture C5E on gemcitabine treatment in PANC1 cells. Mol. Med. Rep..

[22] ICH Q2 (R1) (1997).

[23] Fan Z., Wang C., Yang T., Gao T., Wang D., Zhao X., Guo X., Li D. (2022). Coffee peel extracts ameliorate non-alcoholic fatty liver disease via a fibroblast growth factor 21-adiponectin signaling pathway. Food Funct..

[24] Meng W., Zhao Z., Chen L., Lin S., Zhang Y., He J., Ouyang K., Wang W. (2022). Total flavonoids from chimonanthus nitens oliv. Leaves ameliorate HFD-induced NAFLD by regulating the gut-liver Axis in mice. Foods.

[25] Kandeil M.A., Hashem R.M., Mahmoud M.O., Hetta M.H., Tohamy M.A. (2019). Zingiber officinale extract and omega-3 fatty acids ameliorate endoplasmic reticulum stress in a nonalcoholic fatty liver rat model. J. Food Biochem..

[26] Li H., Chen X., Liu J., Chen M., Huang M., Huang G., Chen X., Du Q., Su J., Lin R. (2021). Ethanol extract of Centella asiatica alleviated dextran sulfate sodium-induced colitis: restoration on mucosa barrier and gut microbiota homeostasis. J. Ethnopharmacol..

[27] Raza S., Rajak S., Upadhyay A., Tewari A., Anthony Sinha R. (2021). Current treatment paradigms and emerging therapies for NAFLD/NASH. Front Biosci (Landmark Ed).

[28] Yang X.F., Lu M., You L., Gen H., Yuan L., Tian T., Li C.Y., Xu K., Hou J., Lei M. (2021). Herbal therapy for ameliorating nonalcoholic fatty liver disease via rebuilding the intestinal microecology. Chin. Med..

[29] Xu X., Poulsen K.L., Wu L., Liu S., Miyata T., Song Q., Wei Q., Zhao C., Lin C., Yang J. (2022). Targeted therapeutics and novel signaling pathways in non-alcohol-associated fatty liver/steatohepatitis (NAFL/NASH). Signal Transduct Target Ther.

[30] Kasper P., Martin A., Lang S., Kutting F., Goeser T., Demir M., Steffen H.M. (2021). NAFLD and cardiovascular diseases: a clinical review. Clin. Res. Cardiol..

[31] Luo H., Wang X., Song S., Wang Y., Dan Q., Ge H. (2022). Targeting stearoyl-coa desaturase enhances radiation induced ferroptosis and immunogenic cell death in esophageal squamous cell carcinoma. OncoImmunology.

[32] Poornima M.S., Sindhu G., Billu A., Sruthi C.R., Nisha P., Gogoi P., Baishya G., GR K. (2022). Pretreatment of hydroethanolic extract of Dillenia indica L. attenuates oleic acid induced NAFLD in HepG2 cells via modulating SIRT-1/p-LKB-1/AMPK, HMGCR & PPAR-alpha signaling pathways. J. Ethnopharmacol..

[33] Cominguez D.C., Park Y.J., Kang Y.M., Nugroho A., Kim S., An H.J. (2022). Clitorin ameliorates western diet-induced hepatic steatosis by regulating lipogenesis and fatty acid oxidation in vivo and in vitro. Sci. Rep..

[34] Li J., Wang T., Liu P., Yang F., Wang X., Zheng W., Sun W. (2021). Hesperetin ameliorates hepatic oxidative stress and inflammation via the PI3K/AKT-Nrf2-ARE pathway in oleic acid-induced HepG2 cells and a rat model of high-fat diet-induced NAFLD. Food Funct..

[35] Carnuta M.G., Deleanu M., Barbalata T., Toma L., Raileanu M., Sima A.V., Stancu C.S. (2018). Zingiber officinale extract administration diminishes steroyl-CoA desaturase gene expression and activity in hyperlipidemic hamster liver by reducing the oxidative and endoplasmic reticulum stress. Phytomedicine.

[36] Kumari S., Deori M., Elancheran R., Kotoky J., Devi R. (2016). In vitro and in vivo antioxidant, anti-hyperlipidemic properties and chemical characterization of Centella asiatica (L.) extract. Front. Pharmacol..

[37] Lv Y., Gao X., Luo Y., Fan W., Shen T., Ding C., Yao M., Song S., Yan L. (2019). Apigenin ameliorates HFD-induced NAFLD through regulation of the XO/NLRP3 pathways. J. Nutr. Biochem..

[38] Lian C.Y., Zhai Z.Z., Li Z.F., Wang L. (2020). High fat diet-triggered non-alcoholic fatty liver disease: a review of proposed mechanisms. Chem. Biol. Interact..

[39] Faran S.A., Asghar S., Khalid S.H., Khan I.U., Asif M., Khalid I., Gohar U.F., Hussain T. (2019). Hepatoprotective and renoprotective properties of lovastatin-loaded ginger and garlic Oil nanoemulsomes: insights into serum biological parameters. Medicina (Kaunas).

[40] Wan X., Li T., Liu D., Chen Y., Liu Y., Liu B., Zhang H., Zhao C. (2018). Effect of marine microalga chlorella pyrenoidosa ethanol extract on lipid metabolism and gut microbiota composition in high-fat diet-fed rats. Mar. Drugs.

[41] Liang S., Zhang Y., Deng Y., He Y., Liang Y., Liang Z., Chen Y., Tang K., Chen R., Yang Q. (2018). The potential effect of Chinese herbal formula hongqijiangzhi fang in improving NAFLD: focusing on NLRP3 inflammasome and gut microbiota. Evid Based Complement Alternat Med 2018.

[42] Alshawsh M.A., Alsalahi A., Alshehade S.A., Saghir S.A.M., Ahmeda A.F., Al Zarzour R.H., Mahmoud A.M. (2022). A comparison of the gene expression profiles of non-alcoholic fatty liver disease between animal models of a high-fat diet and methionine-choline-deficient diet. Molecules.

[43] Rashid M.M., Rahman M.A., Islam M.S., Hossen M.A., Ahmed A.M.A., Afroze M., Habib A.H., Mansoury M.M.S., Alharbi H.F., Algheshairy R.M., Alelwani W., Alnajeebi A.M., Tangpong J., Saha S., Qadhi A., Azhar W. (2022). Natural compounds of lasia spinosa (L.) stem potentiate antidiabetic actions by regulating diabetes and diabetes-related biochemical and cellular indexes. Pharmaceuticals.

[44] Li X., Cheng Y., Li J., Liu C., Qian H., Zhang G. (2022). Torularhodin alleviates hepatic dyslipidemia and inflammations in high-fat diet-induced obese mice via PPARalpha signaling pathway. Molecules.

[45] Kim S.H., Sung M.J., Park J.H., Yang H.J., Hwang J.T. (2013). Boehmeria nivea stimulates glucose uptake by activating peroxisome proliferator-activated receptor gamma in C2C12 cells and improves glucose intolerance in mice fed a high-fat diet. Evid Based Complement Alternat Med.

[46] Nwozo S.O., Osunmadewa D.A., Oyinloye B.E. (2014). Anti-fatty liver effects of oils from Zingiber officinale and Curcuma longa on ethanol-induced fatty liver in rats. J Integr Med.

[47] Tan S.C., Rajendran R., Bhattamisra S.K., Krishnappa P., Davamani F., Chitra E., Ambu S., Furman B., Candasamy M. (2023). Effect of madecassoside in reducing oxidative stress and blood glucose in streptozotocin-nicotinamide-induced diabetes in rats. J. Pharm. Pharmacol..

[48] Jang Y.S., Wang Z., Lee J.M., Lee J.Y., Lim S.S. (2016). Screening of Korean natural products for anti-adipogenesis properties and isolation of kaempferol-3-O-rutinoside as a potent anti-adipogenetic compound from solidago virgaurea. Molecules.

[49] Zhang L., Li H.Z., Gong X., Luo F.L., Wang B., Hu N., Wang C.D., Zhang Z., Wan J.Y. (2010). Protective effects of Asiaticoside on acute liver injury induced by lipopolysaccharide/D-galactosamine in mice. Phytomedicine.

[50] Liu Y., Li D., Wang S., Peng Z., Tan Q., He Q., Wang J. (2023). 6-Gingerol ameliorates hepatic steatosis, inflammation and oxidative stress in high-fat diet-fed mice through activating LKB1/AMPK signaling. Int. J. Mol. Sci..

